# The genome sequence of the white-footed hoverfly, *Platycheirus albimanus* (Fabricius, 1781)

**DOI:** 10.12688/wellcomeopenres.20494.1

**Published:** 2023-12-14

**Authors:** Liam M. Crowley, Katie J. Woodcock, University of Oxford and Wytham Woods Genome Acquisition Lab, Darwin Tree of Life Barcoding collective, Wellcome Sanger Institute Tree of Life Management, Samples and Laboratory team, Wellcome Sanger Institute Scientific Operations: Sequencing Operations, Wellcome Sanger Institute Tree of Life Core Informatics team, Tree of Life Core Informatics collective, Darwin Tree of Life Consortium

**Affiliations:** 1 Department of Biology, University of Oxford, Oxford, England, UK; 2 Tree of Life, Wellcome Sanger Institute, Hinxton, England, UK

**Keywords:** Platycheirus albimanus, white-footed hoverfly, genome sequence, chromosomal, Diptera

## Abstract

We present a genome assembly from an individual female *Platycheirus albimanus* (the white-footed hoverfly; Arthropoda; Insecta; Diptera; Syrphidae). The genome sequence is 677.8 megabases in span. Most of the assembly is scaffolded into 4 chromosomal pseudomolecules, including the X sex chromosome. The mitochondrial genome has also been assembled and is 18.17 kilobases in length. Gene annotation of this assembly on Ensembl identified 12,568 protein coding genes.

## Species taxonomy

Eukaryota; Metazoa; Eumetazoa; Bilateria; Protostomia; Ecdysozoa; Panarthropoda; Arthropoda; Mandibulata; Pancrustacea; Hexapoda; Insecta; Dicondylia; Pterygota; Neoptera; Endopterygota; Diptera; Brachycera; Muscomorpha; Eremoneura; Cyclorrhapha; Aschiza; Syrphoidea; Syrphidae; Syrphinae; Melanostomini; *Platycheirus*; *Platycheirus albimanus* (Fabricius, 1781) (NCBI:txid414846).

## Background

*Platycheirus albimanus* (Fabricius, 1781), also referred to as the white-footed hoverfly, is one of the most familiar, widespread and abundant hoverflies across the UK and Ireland ( Ball & Morris, 2000; Ball & Morris, 2015; Stubbs & Falk, 2002). *Platycheirus* is the second largest British hoverfly genus currently comprising 25 species. *P. albimanus* can be mistaken for other members of the genus including *P. sticticus* and *P. discimanus*. Consequently, use of a specialist key is often necessary to conclusively identify individuals to species level ( Ball & Morris, 2000; Stubbs & Falk, 2002; van Veen, 2014). Adult female *P. albimanus* are identified through the presence and shape of silver-grey abdominal tergite spots, predominantly yellow legs and a faintly dusted face ( Ball & Morris, 2015; Stubbs & Falk, 2002). In males, the colouring of tergite spots is usually bronze but sometimes appears grey or dull yellow. Males also have distinctive tangled hairs at the base of the front femur and a marked swelling at the apex of the front tibia ( Stubbs & Falk, 2002; van Veen, 2014).

It is a relatively small hoverfly species often associated with brambles and nettles in woodland-edges, hedgerows and gardens ( Ball & Morris, 2000; Stubbs & Falk, 2002). The species has multiple broods per season and can be found all year round with numbers peaking twice annually during May to June and July to August ( Ball & Morris, 2015; Stubbs & Falk, 2002). The larvae of *P. albimanus* are aphid predators and have been observed among low-growing foliage as well as on fir trees and common reeds ( Stubbs & Falk, 2002).

Generation of a reference genome for *Platycheirus albimanus* provides a valuable tool to further the knowledge of this prominent UK hoverfly species. Here we present a chromosomally complete genome sequence for *Platycheirus albimanus*, sequenced as part of the Darwin Tree of Life Project, a collaborative effort to sequence all named eukaryotic species in the Atlantic Archipelago of Britain and Ireland.

## Genome sequence report

**Figure 1.**
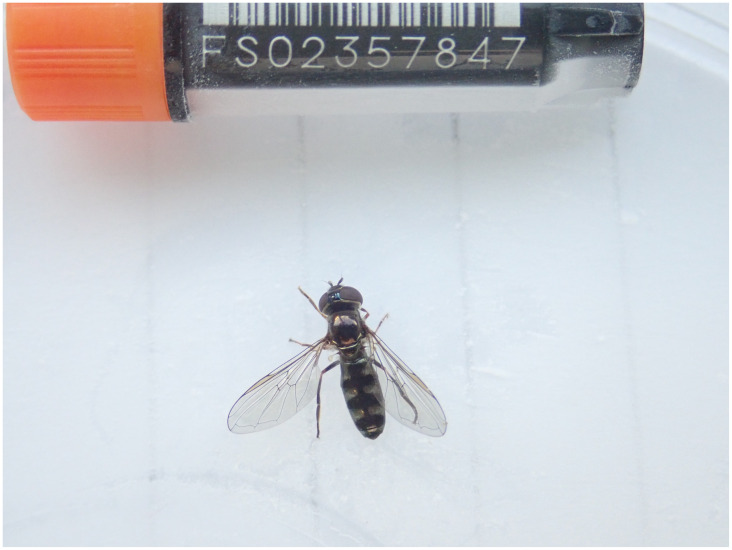
Photograph of the *Platycheirus albimanus* (idPlaAlba1) specimen used for genome sequencing.

The genome was sequenced from one female *Platycheirus albimanus* ( Figure 1) collected from Wytham woods, Oxfordshire, UK (51.77, –1.34). A total of 50-fold coverage in Pacific Biosciences single-molecule HiFi long reads and 27-fold coverage in 10X Genomics read clouds were generated. Primary assembly contigs were scaffolded with chromosome conformation Hi-C data. Manual assembly curation corrected 502 missing joins or mis-joins and removed 10 haplotypic duplications, reducing the assembly length by 1.22% and the scaffold number by 76.73%, and increasing the scaffold N50 by 363.49%.

**Figure 2.**
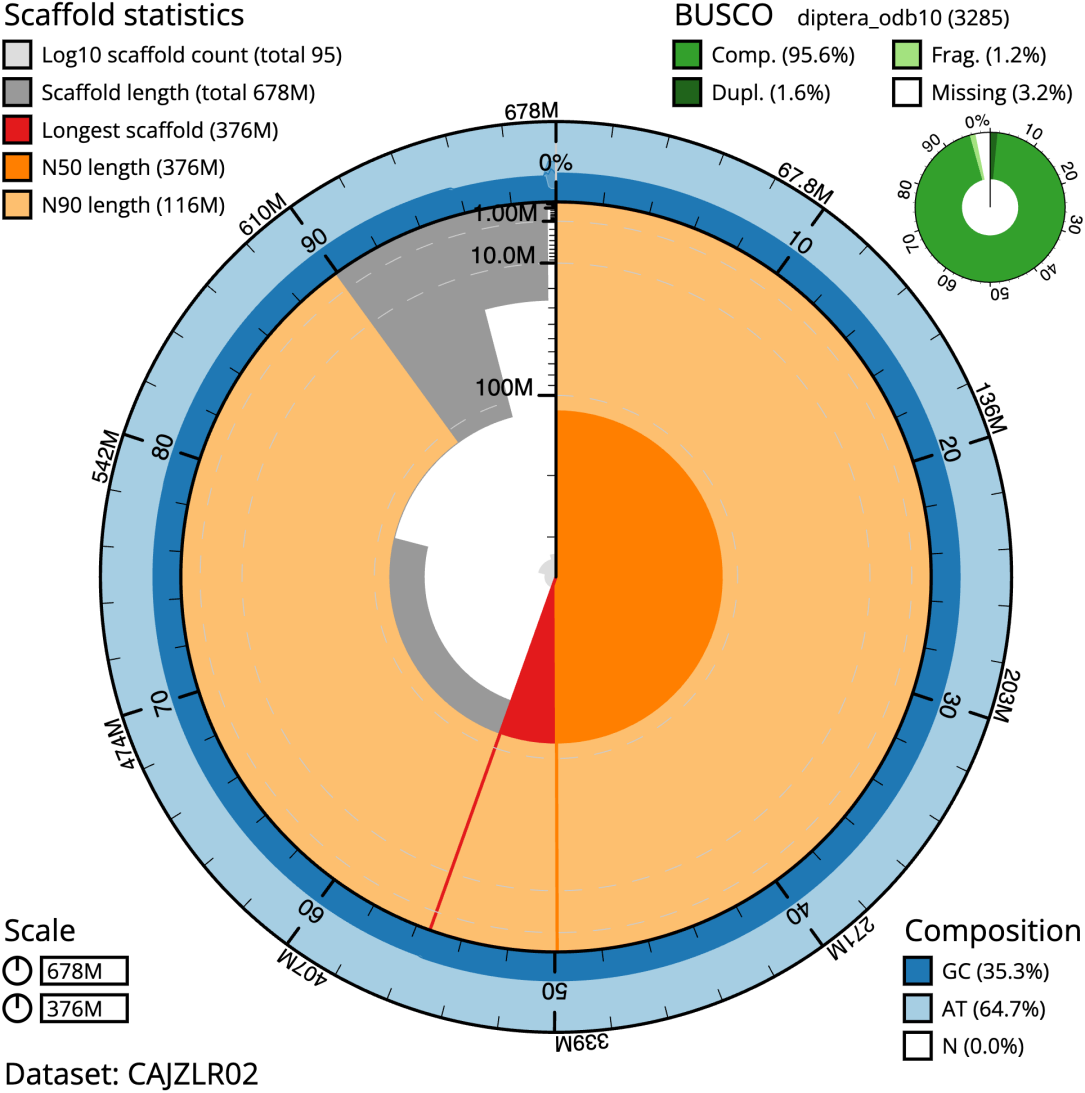
Genome assembly of *Platycheirus albimanus*, idPlaAlba1.2: metrics. The BlobToolKit Snailplot shows N50 metrics and BUSCO gene completeness. The main plot is divided into 1,000 size-ordered bins around the circumference with each bin representing 0.1% of the 677,786,980 bp assembly. The distribution of scaffold lengths is shown in dark grey with the plot radius scaled to the longest scaffold present in the assembly (375,708,846 bp, shown in red). Orange and pale-orange arcs show the N50 and N90 scaffold lengths (375,708,846 and 116,005,501 bp), respectively. The pale grey spiral shows the cumulative scaffold count on a log scale with white scale lines showing successive orders of magnitude. The blue and pale-blue area around the outside of the plot shows the distribution of GC, AT and N percentages in the same bins as the inner plot. A summary of complete, fragmented, duplicated and missing BUSCO genes in the diptera_odb10 set is shown in the top right. An interactive version of this figure is available at https://blobtoolkit.genomehubs.org/view/Platycheirus%20albimanus/dataset/CAJZLR02/snail.

**Figure 3.**
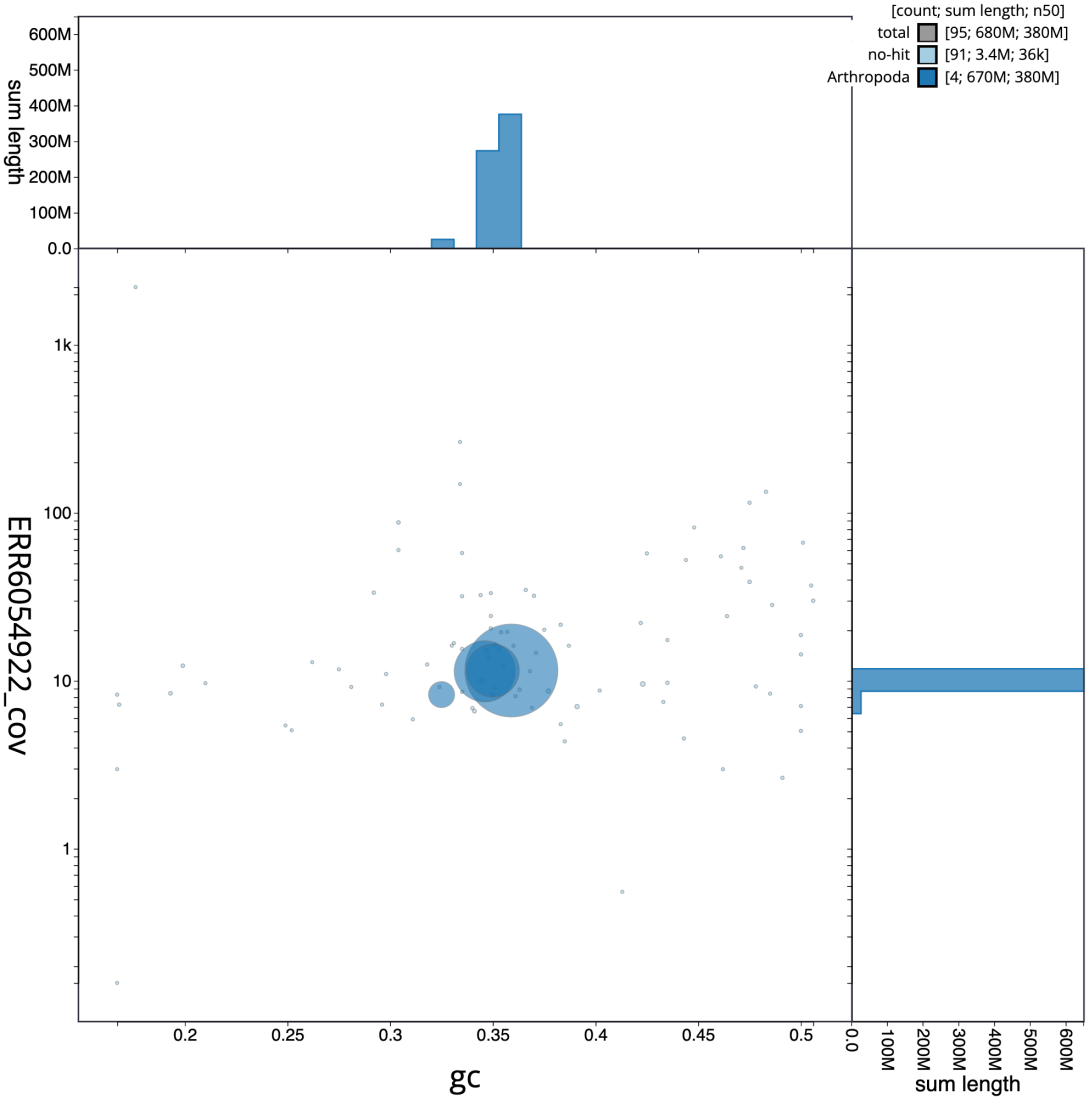
Genome assembly of *Platycheirus albimanus*, idPlaAlba1.2: BlobToolKit GC-coverage plot. Scaffolds are coloured by phylum. Circles are sized in proportion to scaffold length. Histograms show the distribution of scaffold length sum along each axis. An interactive version of this figure is available at https://blobtoolkit.genomehubs.org/view/Platycheirus%20albimanus/dataset/CAJZLR02/blob.

**Figure 4.**
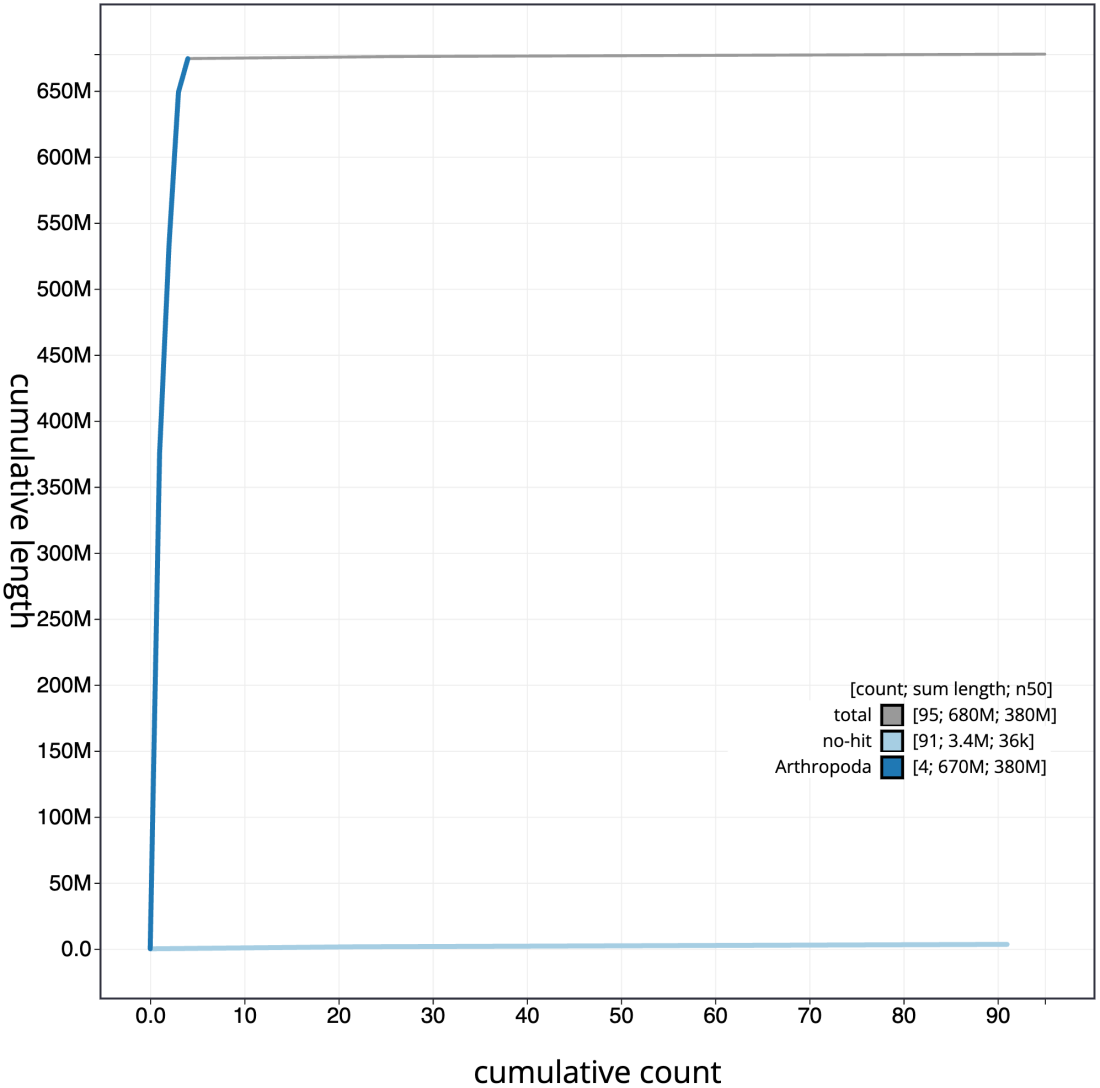
Genome assembly of *Platycheirus albimanus*, idPlaAlba1.2: BlobToolKit cumulative sequence plot. The grey line shows cumulative length for all scaffolds. Coloured lines show cumulative lengths of scaffolds assigned to each phylum using the buscogenes taxrule. An interactive version of this figure is available at https://blobtoolkit.genomehubs.org/view/Platycheirus%20albimanus/dataset/CAJZLR02/cumulative.

**Figure 5.**
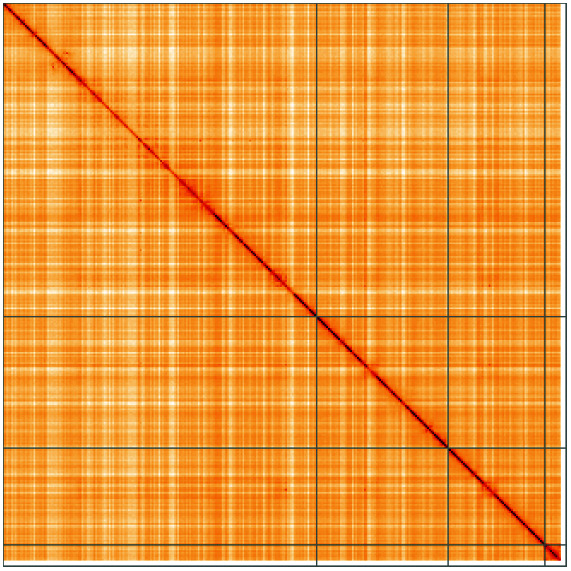
Genome assembly of *Platycheirus albimanus*, idPlaAlba1.2: Hi-C contact map of the idPlaAlba1.2 assembly, visualised using HiGlass. Chromosomes are shown in order of size from left to right and top to bottom. An interactive version of this figure may be viewed at https://genome-note-higlass.tol.sanger.ac.uk/l/?d=OqJMIWx0Rg6Wz0yJDVxuZw.

The final assembly has a total length of 677.8 Mb in 94 sequence scaffolds with a scaffold N50 of 375.7 Mb ( Table 1). The snailplot in Figure 2 provides a summary of the assembly statistics, while the distribution of assembly scaffolds on GC proportion and coverage is shown in Figure 3. The cumulative assembly plot in Figure 4 shows curves for subsets of scaffolds assigned to different phyla. Most (99.52%) of the assembly sequence was assigned to 4 chromosomal-level scaffolds, representing 3 autosomes and the X sex chromosome. The very large repetitive region of chromosome 1, spanning 119.95 Mb to 193.61 Mb is less certain than the rest of the assembly. It has been assembled to best fit the Hi-C data. Chromosome-scale scaffolds confirmed by the Hi-C data are named in order of size ( Figure 5; Table 2). While not fully phased, the assembly deposited is of one haplotype. Contigs corresponding to the second haplotype have also been deposited. The mitochondrial genome was also assembled and can be found as a contig within the multifasta file of the genome submission.

**Table 1.** Genome data for *Platycheirus albimanus*, idPlaAlba1.2.

Project accession data		
Assembly identifier	idPlaAlba1.2	
Species	*Platycheirus albimanus*	
Specimen	idPlaAlba1	
NCBI taxonomy ID	414846	
BioProject	PRJEB45186	
BioSample ID	SAMEA7520157	
Isolate information	idPlaAlba1, female: head and thorax (DNA sequencing), abdomen (Hi-C data) idPlaAlba2, female: head and thorax (RNA sequencing)	
Assembly metrics *		*Benchmark*
Consensus quality (QV)	53.1	*≥ 50*
*k*-mer completeness	99.98	*≥ 95%*
BUSCO **	C:95.6%[S:94.1%,D:1.6%],F:1.2%,M:3.2%,n:3,285	*C ≥ 95%*
Percentage of assembly mapped to chromosomes		*≥ 95%*
Sex chromosomes	X chromosome	*localised homologous pairs*
Organelles	Mitochondrial genome assembled	*complete single alleles*
Raw data accessions		
PacificBiosciences SEQUEL II	ERR6606792, ERR6606791	
10X Genomics Illumina	ERR6054920, ERR6054921, ERR6054922, ERR6054923	
Hi-C Illumina	ERR6054919	
PolyA RNA-Seq Illumina	ERR6286723	
Genome assembly		
Assembly accession	GCA_916050605.2	
*Accession of alternate haplotype*	GCA_916050315.1	
Span (Mb)	677.8	
Number of contigs	691	
Contig N50 length (Mb)	5.6	
Number of scaffolds	94	
Scaffold N50 length (Mb)	375.7	
Longest scaffold (Mb)	375.7	
Genome annotation		
Number of protein-coding genes	12,568	
Number of non-coding genes	1,700	
Number of gene transcripts	19,954	

* Assembly metric benchmarks are adapted from column VGP-2020 of “Table 1: Proposed standards and metrics for defining genome assembly quality” from Rhie *et al.* (2021). ** BUSCO scores based on the diptera_odb10 BUSCO set using v5.3.2. C = complete [S = single copy, D = duplicated], F = fragmented, M = missing, n = number of orthologues in comparison. A full set of BUSCO scores is available at https://blobtoolkit.genomehubs.org/view/Platycheirus%20albimanus/dataset/CAJZLR02/busco.

**Table 2.** Chromosomal pseudomolecules in the genome assembly of Platycheirus albimanus, idPlaAlba1.

INSDC accession	Chromosome	Length (Mb)	GC%
OU696696.1	1	375.71	36.0
OU696697.1	2	157.31	34.5
OU696698.1	3	116.01	35.0
OU696699.1	X	25.4	32.5
OU696700.1	MT	0.02	18.0

The estimated Quality Value (QV) of the final assembly is 53.1 with *k*-mer completeness of 99.98%, and the assembly has a BUSCO v5.3.2 completeness of 95.6% (single = 94.1%, duplicated = 1.6%), using the diptera_odb10 reference set ( *n* = 3,285).

Metadata for specimens, barcode results, spectra estimates, sequencing runs, contaminants and pre-curation assembly statistics are given at https://links.tol.sanger.ac.uk/species/414846.

## Genome annotation report

The *Platycheirus albimanus* genome assembly (GCA_916050605.2) was annotated using the Ensembl rapid annotation pipeline ( Table 1; https://rapid.ensembl.org/Platycheirus_albimanus_GCA_916050605.2/Info/Index). The resulting annotation includes 19,954 transcribed mRNAs from 12,568 protein-coding and 1,700 non-coding genes.

## Methods

### Sample acquisition and nucleic acid extraction

A female *Platycheirus albimanus* (specimen ID Ox000223, ToLID idPlaAlba1) was collected from Wytham Woods, Oxfordshire (biological vice-county Berkshire), UK (latitude 51.77, longitude –1.34) on 2019-08-28 by netting. The specimen used for RNA sequencing, also a female (specimen ID Ox000240, ToLID idPlaAlba2), was collected from the same location on 2019-09-03. Both specimens were collected and formally identified by Liam Crowley (University of Oxford), and then preserved on dry ice.

High molecular weight (HMW) DNA was extracted at the Tree of Life laboratory, Wellcome Sanger Institute (WSI), following a sequence of core procedures: sample preparation; sample homogenisation; HMW DNA extraction; DNA fragmentation; and DNA clean-up. The idPlaAlba1 sample was weighed and dissected on dry ice (as per the protocol https://dx.doi.org/10.17504/protocols.io.x54v9prmqg3e/v1). The head and thorax of the idPlaAlba1 sample was homogenised using a Nippi Powermasher fitted with a BioMasher pestle, following the protocol at https://dx.doi.org/10.17504/protocols.io.5qpvo3r19v4o/v1. DNA was extracted by means of the HMW DNA Extraction: Automated MagAttract protocol ( https://dx.doi.org/10.17504/protocols.io.kxygx3y4dg8j/v1). HMW DNA was sheared into an average fragment size of 12–20 kb in a Megaruptor 3 system with speed setting 30, following the HMW DNA Fragmentation: Diagenode Megaruptor®3 for PacBio HiFi protocol ( https://dx.doi.org/10.17504/protocols.io.8epv5x2zjg1b/v1). Sheared DNA was purified using solid-phase reversible immobilisation (SPRI) (protocol at https://dx.doi.org/10.17504/protocols.io.kxygx3y1dg8j/v1). In brief, the method employs a 1.8X ratio of AMPure PB beads to sample to eliminate shorter fragments and concentrate the DNA. The concentration of the sheared and purified DNA was assessed using a Nanodrop spectrophotometer and Qubit Fluorometer and Qubit dsDNA High Sensitivity Assay kit. Fragment size distribution was evaluated by running the sample on the FemtoPulse system.

RNA was extracted from head and thorax tissue of idPlaAlba2 using the Automated MagMax™ *mir*Vana protocol ( https://dx.doi.org/10.17504/protocols.io.6qpvr36n3vmk/v1). The RNA concentration was assessed using a Nanodrop spectrophotometer and Qubit Fluorometer using the Qubit RNA Broad-Range (BR) Assay kit. Analysis of the integrity of the RNA was done using the Agilent RNA 6000 Pico Kit and Eukaryotic Total RNA assay.

All wet lab protocols developed by the Tree of Life laboratory are publicly available on protocols.io: https://dx.doi.org/10.17504/protocols.io.8epv5xxy6g1b/v1.

### Sequencing

Pacific Biosciences HiFi circular consensus and 10X Genomics read cloud DNA sequencing libraries were constructed according to the manufacturers’ instructions. Poly(A) RNA-Seq libraries were constructed using the NEB Ultra II RNA Library Prep kit. DNA and RNA sequencing was performed by the Scientific Operations core at the WSI on Pacific Biosciences SEQUEL II (HiFi), Illumina HiSeq 4000 (RNA-Seq) and HiSeq X Ten (10X) instruments. Hi-C data were also generated from abdomen tissue of idPlaAlba1 using the Arima2 kit and sequenced on the HiSeq X Ten instrument.

### Genome assembly, curation and evaluation

Assembly was carried out with Hifiasm ( Cheng *et al.*, 2021) and haplotypic duplication was identified and removed with purge_dups ( Guan *et al.*, 2020). One round of polishing was performed by aligning 10X Genomics read data to the assembly with Long Ranger ALIGN, calling variants with FreeBayes ( Garrison & Marth, 2012). The assembly was then scaffolded with Hi-C data ( Rao *et al.*, 2014) using SALSA2 ( Ghurye *et al.*, 2019). The assembly was checked for contamination and corrected using the gEVAL system ( Chow *et al.*, 2016) as described previously ( Howe *et al.*, 2021). Manual curation was performed using gEVAL, HiGlass ( Kerpedjiev *et al.*, 2018) and Pretext ( Harry, 2022). The mitochondrial genome was assembled using MitoHiFi ( Uliano-Silva *et al.*, 2023), which runs MitoFinder ( Allio *et al.*, 2020) or MITOS ( Bernt *et al.*, 2013) and uses these annotations to select the final mitochondrial contig and to ensure the general quality of the sequence.

A Hi-C map for the final assembly was produced using bwa-mem2 ( Vasimuddin *et al.*, 2019) in the Cooler file format ( Abdennur & Mirny, 2020). To assess the assembly metrics, the *k*-mer completeness and QV consensus quality values were calculated in Merqury ( Rhie *et al.*, 2020). This work was done using Nextflow ( Di Tommaso *et al.*, 2017) DSL2 pipelines “sanger-tol/readmapping” ( Surana *et al.*, 2023a) and “sanger-tol/genomenote” ( Surana *et al.*, 2023b). The genome was analysed within the BlobToolKit environment ( Challis *et al.*, 2020) and BUSCO scores ( Manni *et al.*, 2021; Simão *et al.*, 2015) were calculated.

Table 3 contains a list of relevant software tool versions and sources.

**Table 3.** Software tools: versions and sources.

Software tool	Version	Source
BlobToolKit	4.1.7	https://github.com/blobtoolkit/blobtoolkit
BUSCO	5.3.2	https://gitlab.com/ezlab/busco
FreeBayes	1.3.1-17-gaa2ace8	https://github.com/freebayes/freebayes
gEVAL	N/A	https://geval.org.uk/
Hifiasm	0.12	https://github.com/chhylp123/hifiasm
HiGlass	1.11.6	https://github.com/higlass/higlass
Long Ranger ALIGN	2.2.2	https://support.10xgenomics.com/genome-exome/ software/pipelines/latest/advanced/other-pipelines
Merqury	MerquryFK	https://github.com/thegenemyers/MERQURY.FK
MitoHiFi	2	https://github.com/marcelauliano/MitoHiFi
PretextView	0.2	https://github.com/wtsi-hpag/PretextView
purge_dups	1.2.3	https://github.com/dfguan/purge_dups
SALSA	2.2	https://github.com/salsa-rs/salsa
sanger-tol/genomenote	v1.0	https://github.com/sanger-tol/genomenote
sanger-tol/readmapping	1.1.0	https://github.com/sanger-tol/readmapping/tree/1.1.0

### Genome annotation

The Ensembl gene annotation system ( Aken *et al.*, 2016) was used to generate annotation for the *Platycheirus albimanus* assembly (GCA_916050605.2). Annotation was created primarily through alignment of transcriptomic data to the genome, with gap filling via protein-to-genome alignments of a select set of proteins from UniProt ( UniProt Consortium, 2019).

### Wellcome Sanger Institute – Legal and Governance

The materials that have contributed to this genome note have been supplied by a Darwin Tree of Life Partner. The submission of materials by a Darwin Tree of Life Partner is subject to the **‘Darwin Tree of Life Project Sampling Code of Practice’**, which can be found in full on the Darwin Tree of Life website here. By agreeing with and signing up to the Sampling Code of Practice, the Darwin Tree of Life Partner agrees they will meet the legal and ethical requirements and standards set out within this document in respect of all samples acquired for, and supplied to, the Darwin Tree of Life Project.

Further, the Wellcome Sanger Institute employs a process whereby due diligence is carried out proportionate to the nature of the materials themselves, and the circumstances under which they have been/are to be collected and provided for use. The purpose of this is to address and mitigate any potential legal and/or ethical implications of receipt and use of the materials as part of the research project, and to ensure that in doing so we align with best practice wherever possible. The overarching areas of consideration are:

•   Ethical review of provenance and sourcing of the material

•   Legality of collection, transfer and use (national and international)

Each transfer of samples is further undertaken according to a Research Collaboration Agreement or Material Transfer Agreement entered into by the Darwin Tree of Life Partner, Genome Research Limited (operating as the Wellcome Sanger Institute), and in some circumstances other Darwin Tree of Life collaborators.

## Data Availability

European Nucleotide Archive: *Platycheirus albimanus* (white-footed hoverfly). Accession number PRJEB45186; https://identifiers.org/ena.embl/PRJEB45186 ( Wellcome Sanger Institute, 2022). The genome sequence is released openly for reuse. The *Platycheirus albimanus* genome sequencing initiative is part of the Darwin Tree of Life (DToL) project. All raw sequence data and the assembly have been deposited in INSDC databases. Raw data and assembly accession identifiers are reported in Table 1.

## Author information

Members of the University of Oxford and Wytham Woods Genome Acquisition Lab are listed here: https://doi.org/10.5281/zenodo.7125292.

Members of the Darwin Tree of Life Barcoding collective are listed here: https://doi.org/10.5281/zenodo.4893703.

Members of the Wellcome Sanger Institute Tree of Life Management, Samples and Laboratory team are listed here: https://doi.org/10.5281/zenodo.10066175.

Members of Wellcome Sanger Institute Scientific Operations: Sequencing Operations are listed here: https://doi.org/10.5281/zenodo.10043364.

Members of the Wellcome Sanger Institute Tree of Life Core Informatics team are listed here: https://doi.org/10.5281/zenodo.10066637.

Members of the Tree of Life Core Informatics collective are listed here: https://doi.org/10.5281/zenodo.5013541.

Members of the Darwin Tree of Life Consortium are listed here: https://doi.org/10.5281/zenodo.4783558.
