# Supplementary material for: The genome sequence of the white-footed hoverfly, Platycheirus albimanus (Fabricius, 1781)
Source: Wellcome Open Res. 2023 Dec 14;8:572. [Version 1] doi: 10.12688/wellcomeopenres.20494.1 (PMC11148528; doi:10.12688/wellcomeopenres.20494.1)
Supplement: Peer review history

# Reviewer report: Reviewer response for version 1

**DOI:** 10.21956/wellcomeopenres.22687.r73171

**Published:** 2024-06-03

**Authors:** Eric Aguiar

**Affiliations:** 1 Universidade Estadual de Santa Cruz, Ilhéus, State of Bahia, Brazil

The manuscript follows a series of articles regarding insect genomes within a consortium supported by the Sanger Institute. They have reached a selection of important species to sequence and a robust methodology surrounded by cutting-edge deep sequencing apparatus. Thus, since it is a well-written Data manuscript with both methods and results correct and concise.

Are sufficient details of methods and materials provided to allow replication by others?

Yes

Is the rationale for creating the dataset(s) clearly described?

Yes

Are the datasets clearly presented in a useable and accessible format?

Yes

Are the protocols appropriate and is the work technically sound?

Yes

Reviewer Expertise:

Bioinformatics

I confirm that I have read this submission and believe that I have an appropriate level of expertise to confirm that it is of an acceptable scientific standard.

# Reviewer report: Reviewer response for version 1

**DOI:** 10.21956/wellcomeopenres.22687.r81586

**Published:** 2024-05-29

**Authors:** Ana Paula Zotta Mota

**Affiliations:** 1 Institut Sophia Agrobiotech, INRAE, CNRS, University of Côte d'Azur, Nice, Provence-Alpes-Côte d'Azur, France

The Darwin Tree of Life initiative and the production of high-quality genome assemblies represent a crucial first step towards gaining a deeper understanding of evolutionary processes. The data presented in this paper are excellent, demonstrating high coverage of the genome and exceptional data quality. The methods used are sound and appropriate for the type of data and the results obtained.

 I have a few suggestions that could further enhance the findings. In the Hi-C map, the boundaries between chromosomes are not clearly visible. The authors might consider adjusting the resolution or color scale based on previous feedback to improve visibility.

 Additionally, I wonder if the authors have searched for canonical telomeric sequences (TTAGGG) within their genome assembly. Highlighting these sequences and their positions on the Hi-C map, perhaps using a wig file to indicate the locations of these telomeric repeats, could be very informative. This addition would complement the already impressive results and provide clearer insights into chromosome organization.

 Another suggestion is to provide information about the expected genome size and the size obtained, as well as the number of chromosomes, possibly drawing comparisons with closely related species.

 In conclusion, this is an excellent genome assembly, and these few adjustments would further enhance the description of this species and contribute valuable knowledge to the scientific community. I appreciate the initiative's commitment to open data.

Are sufficient details of methods and materials provided to allow replication by others?

Yes

Is the rationale for creating the dataset(s) clearly described?

Yes

Are the datasets clearly presented in a useable and accessible format?

Yes

Are the protocols appropriate and is the work technically sound?

Yes

Reviewer Expertise:

I work on the genome of plant-parasitic nematodes from the genus Meloidogyne. I have assembled and helped on the genome of different species of plant parasitic nematodes, which have a complex allopolyploid genome.

I confirm that I have read this submission and believe that I have an appropriate level of expertise to confirm that it is of an acceptable scientific standard, however I have significant reservations, as outlined above.

# Reviewer report: Reviewer response for version 1

**DOI:** 10.21956/wellcomeopenres.22687.r74297

**Published:** 2024-03-01

**Authors:** De-Long Guan

**Affiliations:** 1 Shaanxi Normal University, Xi'an, Shaanxi, China

I am glad to see your article on the genome assembly of *Platycheirus albimanus*. I appreciate the time and effort you put into this work. Genome sequencing is crucial for advancing our scientific understanding, and projects like yours are helping to make whole genome data more accessible.

 Overall, I found your manuscript to be well written and the methods sound. I was able to follow your assembly and annotation processes without difficulty. The quality of assembly you achieved is impressive, especially the level of chromosomal scaffolding. I also thought the figures were well-designed to highlight the key results.

 One small point of feedback relates to the HiC contact map figure. While the overall contacts between scaffolds are clear, I had a little trouble distinguishing the individual chromosomes due to the density of interactions shown. A potential way to address this could be adjusting the resolution or color scale to better delineate each pseudomolecule.

 In closing, I believe this genome will serve as a valuable reference for future research on *P. albimanus* and Dipteran insects more broadly. Please address the minor comment above as you are able. Subject to satisfactory revision. Thank you again for your contribution to open data.

Are sufficient details of methods and materials provided to allow replication by others?

Yes

Is the rationale for creating the dataset(s) clearly described?

Yes

Are the datasets clearly presented in a useable and accessible format?

Yes

Are the protocols appropriate and is the work technically sound?

Yes

Reviewer Expertise:

I focus on the phylogeny and evolution of insects, especially on grasshoppers. Since 2016, I have personally helped generate several genome sequences across a diverse array of insect species. Through this long-term involvement, I have developed a thorough understanding of the methodological approaches, bioinformatic workflows, and computational techniques employed in whole genome projects.

I confirm that I have read this submission and believe that I have an appropriate level of expertise to confirm that it is of an acceptable scientific standard.
